# Association of Placental Pathology and antibiotic exposure after birth with the Severity of Necrotizing Enterocolitis in Preterm infants - A Matched Case-Control Study

**DOI:** 10.21203/rs.3.rs-5717937/v1

**Published:** 2025-02-04

**Authors:** Parvesh Mohan Garg, Robin Riddick, Abu Yusuf Ansari, Aubrey rebentisch, Avinash Shetty, Kristin Adams, William B. Hillegass, Padma Garg

**Affiliations:** Wake Forest University; University of Mississippi Medical center; University of Mississippi Medical center; University of Mississippi Medical Center; University of Mississippi Medial Center; University of Mississippi Medial Center; University of Mississippi Medial Center; University of Mississippi Medial Center

## Abstract

**Objective:**

To determine the association between antibiotic exposure following birth and necrotizing enterocolitis (NEC) severity in preterm infants.

**Methods:**

This single center matched case-control study included infants with NEC (n=107) and matched controls (n= 130) with antibiotic exposure =< 3 days and > 3 days after birth.

**Results:**

Out of 212 infants,103 infants (48.5%) received antibiotics =< 3 days, and 109 infants (51.5%) received antibiotics >3 days. On the multivariate regression, Infants receiving antibiotics for >3 day had higher risk for medical NEC (aOR 2.61,95% CI 1.35 −5.16; p=0.005) and surgical NEC (aOR 3.33, CI 1.57–7.40; p=0.02) than controls. In NEC cohort, those receiving antibiotics for >3 days were like to die (OR 7.88,95% CI 1.99- 53.74; p=0.010) than those receiving antibiotics <3 days.

**Conclusion:**

Infants exposed with early antibiotics >3 days after birth were more likely associated with NEC and were at greater risk of death.

## Introduction

Necrotizing enterocolitis (NEC) affecting about 5–10% of preterm infants with a birth weight ≤ 1500 grams [1, 2] and remains a leading cause for surgical intervention, mortality and the health care cost [3, 4]. In preterm infants, NEC is characterized by extensive intestinal necrosis and inflammation [5].

The risk factors associated with NEC are multifactorial, including adverse intrauterine environment (inflammatory and/or hypoxic), and postnatal exposure to antibiotics and the inflammatory stimuli may switch innate immune cells to a sustain a chronic or exaggerated production of pro-inflammatory cytokines leading to increased risk of NEC (double-Hit) in preterm infants [6].The placental pathology provides a unique opportunity to understand the antenatal exposure and is not fully utilized in neonatal care[7, 8]. Published studies have[9] reported that the presence of placental vascular obstructive lesions on the fetal side and histological chorioamnionitis [10] was associated with increased rate of necrotizing enterocolitis. In a systemic review, histological chorioamnionitis with the fetal response ( funisitis and /or fetal vasculitis) was associated with NEC [11]. We have recently reported that preterm infants with medical and surgical NEC had an increased frequency of inflammatory and vascular placental pathologic lesions compared with matched controls [12–14].

Most of preterm infants receive antibiotics after birth. Many reports have shown the association between the antibiotic exposure and the necrotizing enterocolitis [15]. While some studies have demonstrated an increased risk of NEC [16, 17], other studies have demonstrated seemingly contrary findings of decreased NEC with early antibiotics [18, 19]. Studies using animal models have also yielded differing findings of benefit vs. harm of early antibiotic exposure on subsequent NEC susceptibility [20–22]. The clinical combined impact of antibiotic exposure along with placental inflammatory and the vascular lesions on the severity of NEC is not well studied. Therefore, the objectives of this study were to 1) determine the impact on the antibiotic exposure duration ( = < 3 days and > 3 days after birth) on the severity of NEC. 2) To determine the impact of placental lesions determined by using a uniform classification of placental histopathology [23] along with antibiotic duration exposure following birth on the severity of NEC.

## Methods

### Study design

A matched case-control study was conducted at the University of Mississippi Medical Center (UMMC) at Jackson, Mississippi, after approval by the Institutional Review Board (2017 – 0127). UMMC has a level 4 neonatal intensive care unit (NICU), which is a regional referral center for neonates with surgical NEC for the entire state.

### Clinical information

We recorded demographic characteristics, including birth weight, GA, sex, race, and mode of delivery (cesarean delivery vs. vaginal delivery), APGAR scores at 5 minutes and the out born status. We also collected information regarding maternal factors, including pregnancy-induced hypertension, chorioamnionitis, and antenatal steroids. Infants who did not show obvious pneumatosis on radiology were classified as ≥ stage II NEC only if they displayed highly suggestive clinical signs with loss of bowel sounds, generalized abdominal tenderness, abdominal distension, systemic instability with increased needs for respiratory and hemodynamic support, and radiological evidence of intestinal dilatation, fixed bowel loops and/or portal venous gas on abdominal X-ray.

We also gathered information on the length of stay defined as the total duration of hospitalization from the day of admission until discharge or death and mortality defined as death due to any cause before hospital discharge.

#### NEC information

NEC was defined using Bell’s criteria[24], and Bell stage III/surgical NEC frequency was gathered[24]. Age at NEC diagnosis (days of life) and abdominal X-ray findings were also collected.

#### Postoperative Information

Postoperative information such as total parenteral nutrition days (TPN), time to full feeds (≥ 120 ml/kg/day), postoperative ileus days (number of days infants not fed following surgery) were also collected.

#### Antibiotic exposure Information

Following birth, at UMMC, the infants receive ampicillin and gentamicin as the first line of antibiotics for suspect early onset sepsis after sending the blood culture. The duration of antibiotics was guided by the infant clinical condition, antenatal history and the service provider. For our study, the infants were divided into two groups who received antibiotics = < 3 days and > 3 days of intravenous antibiotics.

#### Brain Injury Information

Brain MRI without contrast was obtained at the term equivalent age and scored independently by two pediatric radiologists using a Woodward et al. scoring system of eight scales for white and gray matter injury [25].

### Placental Pathology

A board-certified pathologist routinely examines placentas from all preterm births at our institute for gross and histologic findings. The umbilical cord, membranes, and placental disc are inspected for any gross abnormalities. The placenta is weighed after removing the umbilical cord, fetal membranes, and non-adherent blood clots. Placentas weighing below the 10th percentile for estimated GA were considered small for GA, and those weighing > 90th percentile for GA were considered large for GA [26]. The placental disk is then serially sectioned at 1–2-cm intervals and examined for intraparenchymal lesions. Representative sections of the umbilical cord, fetal membranes, placental parenchyma, and any abnormalities seen on gross examination are submitted for standard histological examination.

Based on the Amsterdam criteria, the histopathologic abnormalities assessed were divided into the following subcategories [23].

1. Acute histologic chorioamnionitis with or without fetal inflammatory response.
2. Chronic Villitis with or without obliterative fetal vasculopathy.
3. Maternal vascular malperfusion (MVM) including maternal vascular lesions (decidual arteriopathy, mural hypertrophy, incomplete transformation of spiral arteries, and decidual necrosis), infarcts, hemorrhage or hematoma, thrombi, villous changes (chorangiosis, syncytial knots, distal villous hypoplasia, and accelerated villous maturation), and placental hypoplasia [27–29].
4. Fetal vascular malperfusion (FVM).
5. Other inflammatory lesions (chronic deciduitis with plasma cells, massive chronic intervillositis, perivillous fibrin, and histiocystic intervillositis), delayed villous maturation, and villous edema [30].

The presence of more than one of the defined placental lesions was classified as multiple placental pathologies. A representative set of placentas (n = 30) were randomly chosen by a pediatric pathologist, who reassessed the histology while blinded to the placental reports, clinical history, and the outcomes. This was matched with the pathology reports and resulted in 93% concordance. The random review of 30 placentas was done to validate the initial pathologist’s reading.

#### Outcome

The severity of NEC was defined as infants without NEC, NEC treated medically, and those with NEC requiring surgery.

### Statistical Methods

For normally distributed continuous variables, we summarized the data as means with standard deviation (± SD), and comparisons between groups were performed using Student’s t-test and ANOVA. For continuous data exhibiting skewed distribution, differences were tested using the Mann-Whitney U test or Kruskal-Walli’s test. Categorical data were summarized as counts with relative frequencies as percentages, and differences among the groups were analyzed using the Chi-squared test (*χ*^2^ test) or Fisher’s exact test. Multivariate logistic regression analysis was done to assess the association between antibiotic exposure and the severity of NEC. A 2-sided *p*-value < 0.05 was considered statistically significant for all the analyses. All the statistical analyses were performed in R statistical software (version 4.03; The R Foundation for Statistical Computing).

## Results

### Antibiotics exposure following birth and NEC severity

#### Clinical information of infants receiving antibiotics = < and > 3 days after birth:

##### Any NEC vs. Controls:

**One** hundred and three infants (n = 103) were included in the analysis receiving antibiotics for less than three days. 33 (33/103, 32%) infants had any NEC (Medical or surgical NEC). Those with any NEC had significantly higher exposure to PIH (36.4% vs 13%, p = 0.014) and stayed in NICU longer (26 days [6.75;69.8] vs. 73[41;126]; p < 0.001) compared to controls.

Out of 33 cases of any NEC, 20/33 (60.6%) had medical NEC and 13/33 (39.3%) had surgical NEC.

###### Medical NEC vs. Surgical NEC vs. controls

Infants with surgical NEC had significantly lower birth weight, lower median gestational age, had higher morality and longer hospital stay compared to infants with medical NEC and the controls. The data are summarized in Table 1 and Table 2.

#### Infants receiving > 3 days of antibiotics:

##### Any NEC vs Controls:

**One** hundred and nine infants (n = 109) were included in the analysis receiving antibiotics for more than three days. 64 (64/109,58.7%) infants had any NEC (Medical or surgical NEC). Those with any NEC had significantly lower median birth weight (735gm [630;1128] vs. 1020gm [780;1680]; p = 0.010) and lower median gestational age (26.5 weeks [24.4;29.7] vs.28.6 weeks [25.4;32]; p = 0.03) than controls. Those with any NEC were exposed to PIH (39.7% vs.4.5%; p < 0.001) more frequently, were SGA (44.3% vs.17.3%; p = 0.08) and had APGAR score less than 6 at 5 mins (35.5% vs 13.6%; p = 0.022) more frequently than controls. Infants receiving antibiotics longer than 3 days after birth had significantly longer length of stay (103 days [64;163) vs. 73 days [30;97]; p = 0.001) and the higher mortality (31.2% vs. 6.6%; p = 0.004) than controls.

Out of 64 cases of any NEC, 34/64 (53%) had medical NEC and the 30/64 (47%) cases had the surgical NEC.

##### Medical NEC vs. Surgical NEC vs. controls:

Those with surgical NEC and medical NEC had significantly lower birth weight, were SGA more frequently, exposed to PIH more frequently, lower APGAR scores less than 6 at 5 minutes, had longer length of stay and higher mortality compared to controls. The data is summarized in Table 2.

**The data are summarized in** Table 1.

##### NEC features and antibiotic exposure duration in surgical NEC cohort:

Forty-three infants with surgical NEC were included in the study. 13/43 (30.3%) infants received antibiotics = < 3days of antibiotics and 30/43(69.7%) received antibiotics > 3days. Those infants receiving antibiotics > 3 days had later median age of NEC onset (18.0 [9.25;34.0] vs. 5.00 [4.00;19.0]; p = 0.002), higher median CRP on the day of NEC onset (2.60 [1.10;6.00] vs. 0.70 [0.50;1.10]; p = 0.003) and longer post operative ileus days 16 days [12.5;21.0] vs. 7 days [7.00;12.0]; p = 0.021) compared those receiving antibiotics = < 3days. The data has been summarized in **supplemental Table 1.**

##### Multi regression analysis:

On the multivariate regression analysis, Infants receiving antibiotics for > 3 days after birth had significantly higher adjusted odds of medical NEC (aOR 2.61,95% CI 1.35–5.16; p = 0.005) and surgical NEC (aOR 3.33, CI 1.57–7.40; p = 0.02) than control group. In NEC cohort, those receiving antibiotics for > 3 days after birth had higher odds of mortality (OR 7.88,95% CI 1.99–53.74; p = 0.010) than those receiving antibiotics less than 3 days (Table 4 and Table 4A).

###### Placental lesions and Antibiotic exposure < 3 days

The data are summarized in Table 3. 103 infants were included in the analysis. Those with surgical NEC had higher exposure to acute histologic chorioamnionitis and chorioamnionitis with the fetal response (61.5% vs 11.4%) and the maternal vascular malformation and CFR (46.2% vs 4.3%) compared to controls. **The data are summarized in** Table 5.

###### Placental lesions and Antibiotic exposure > 3 days

108 infants were included in the analysis. Infants receiving antibiotics more than 3 days had higher AHC + CFR lesions (26.7% vs 5.9%) and higher MVM + CFR lesions (20% vs. 5.9%) on the placental pathology in infants with surgical NEC compared to those with medical NEC. **The data are summarized in** Table 5.

###### The association between placental lesions, antibiotic duration and NEC severity

####### Acute histologic Chorioamnionitis

64 infants with acute histologic chorioamnionitis were included in the analysis. 27/64 (42%) infants received antibiotics = < 3 days and 37/64 (58%) infants received antibiotics for more than 3 days. 19/64 (29.6%) had medical NEC and 21/64 (32.8%) had surgical NEC. Infants receiving antibiotics more than 3 days were more likely at risk of medical (79% vs 38%; p = 0.021) and surgical NEC (62% vs. 38%; p = 0.021).

#### Maternal Vascular Malperfusion:

134 infants with maternal vascular malperfusion were included in the analysis. 66/134 (49%) infants received antibiotics = < 3 days and 68/134 (51%) infants received antibiotics for more than 3 days. 43/134 (32%) had medical NEC and 21/64 (22.3%) had surgical NEC. Infants receiving antibiotics more than 3 days were more likely at risk of medical (60% vs 36%; p = 0.007) and surgical NEC (67% vs. 36%; p = 0.007).

The association between the infants with other placental lesions such as acute histologic chorioamnionitis with fetal response, SGA placenta, antibiotic duration and NEC severity is summarized in Table 6.

## Discussion

Our study shows that infants receiving antibiotics for more than 3 days were likely associated with medical and surgical NEC than controls and were at higher odds of mortality compared to those receiving antibiotics less than 3 days. Those infants receiving antibiotics > 3 days had later median age of NEC onset, higher median CRP on the day of NEC onset and longer post operative ileus days compared to those receiving antibiotics = < 3days in surgical NEC cohort. On placental pathology, infants receiving any antibiotic exposure more than 3 days more likely had combination lesions of AHC + CFR and CFR + MVM and were more likely associated with surgical NEC.

Many reports have shown the association between the antibiotic exposure and the necrotizing enterocolitis [15]. While few reports have demonstrated an increased risk of NEC [16, 17], other studies have demonstrated seemingly contrary findings of decreased NEC with early antibiotics [18, 19].

Studies using animal models have also yielded differing findings of benefit vs. harm of early antibiotic exposure on subsequent NEC susceptibility [20–22].

The antibiotics’ following birth is associated with increased risk of NEC due to changes in gut microbiome, intestinal epithelium maturation and the intestinal barrier function as shown in animal studies [31–34]. A recent metanalysis by Pammi et al had shown that Microbial dysbiosis preceding NEC in preterm infants is characterized by increased relative abundances of Proteobacteria and decreased relative abundances of Firmicutes and Bacteroidetes [35]. Microbiome optimization may provide a novel strategy for preventing NEC. However, a recent randomized, double-blinded placebo-controlled trial failed to demonstrate that withholding 48 hours of standard intravenous antibiotics immediately after birth improved the microbiome or clinical outcomes [36]. In our study, the infants with exposure to acute histologic chorioamnionitis and maternal vascular malformation and receiving antibiotics more than 3 days following birth were more likely associated with medical or surgical NEC. A systemic review noted that histological chorioamnionitis accompanied by a fetal response (funisitis and /or chorionic plate vasculitis) was associated with NEC [11]. Moore and colleagues [10] also noted that infants with NEC (≥ Bells stage II) had an increased risk of placental infarctions and histological chorioamnionitis compared to controls. Dix et al [9] reported that the presence of placental fetal vascular obstructive lesions was associated with increased frequency of NEC in preterm infants, though differences in placental pathologic lesions associated with surgical and medical NEC were not examined. A recent meta-analysis[37] of 33 relevant studies found clinical chorioamnionitis was significantly associated with NEC, but the overall association between histologic chorioamnionitis (HC) and NEC was not statistically significant. In contrast, sub-analysis of HC with fetal involvement was highly associated with NEC. A recently published case-control retrospective study [38] of infants < 33 weeks gestational age or < 1500 grams birthweight evaluated placental pathology for FIR (Fetal inflammatory response) progression and severity defined according to Amsterdam classification. The authors found that both stage and grade of FIR were positively correlated with the stage of NEC (p < 0.001). On multinomial logistic regression, stage III NEC was associated with high stage of FIR (p < 0.001).

Placental inflammation with evidence of a fetal response may place the fetal intestine at greater risk of subsequent injury when exposed to NEC-related stimuli such as antibiotic exposure after birth – a concept which supports the double hit hypothesis for surgical NEC [6]. A fetal inflammatory response in the placenta is defined by the presence of neutrophils within the muscular walls of veins and/or arteries in the chorioamniotic plate and/or umbilical cord [39, 40]. This fetal placental response may be associated with a systemic inflammatory response with an exaggerated pro-inflammatory cytokine activation. Fibrin deposition in response to endothelial damage (vasculitis) in the placental vasculature may be the linking mechanism between chorioamnionitis, the fetal response, and the postnatal intestinal injury [41, 42]. Wolfs et al have recently reported that *IL-1α* mediated chorioamnionitis induces depletion of FoxP3 + cells and ileal inflammation in the ovine fetal gut [43]. In another study by Wolf et al, reported that the distribution of the tight junctional protein ZO-1, the immune defense, and vascular function are all immature in the fetal intestines at lower gestational ages and are further compromised by endotoxin-induced chorioamnionitis in a sheep model [44]. Yan et al has reported in a newborn mice model [45] that prenatal inflammation increases the susceptibility to NEC, downregulates intestinal VEGFR2 signaling, and impairs the perinatal intestinal microvascular development via a TNF mechanism. Their data also suggest that alteration of intestinal microvascular development may be a key mechanism by which premature infants exposed to prenatal inflammation are at risk for NEC and preserving the VEGF/VEGF receptor 2 signaling pathway may help prevent NEC development.

To our knowledge, one of the few studies to report the combined exposure to the placental lesions, postnatal antibiotic exposure and with the severity of NEC in a predominantly African American population, using a uniform classification of placental pathologic lesions. Our study has shown the impact of antibiotics exposure on the NEC presentation and the postoperative outcomes in surgical NEC cohort.

Our findings have few limitations: Our study was a single center and case-control study, and we cannot infer causality, and our findings may not be generalizable to other populations. We had a modest number of participants for our group comparisons. We may be underpowered to detect other associations between pathologic placental findings, antibiotic exposure following birth and NEC.

In conclusion, our study demonstrates that that infants receiving antibiotics for longer duration (> 3 days) were likely associated with necrotizing enterocolitis and were at higher odds of mortality. In our data, infants exposed to placental inflammatory and vascular lesions and antibiotics for more than 3 days following birth were likely associated with medical and surgical NEC supporting the double hit model. Prospective clinical, translational human and animal studies are greatly to fully understand the combined impact of placental lesions and postnatal antibiotic exposure on the neonatal morbidities and mortality to improve the outcomes. Optimizing antibiotic duration following birth is greatly needed to have developmentally appropriate gut maturation and adaptation.

## Figures and Tables

**Table 1:** Clinical information in controls and infants with any NEC

	=<3days antibiotics				>3 days antibiotics			
	[ALL] N=103	Any NEC N=33	Control N=70	p	[ALL] N=109	Any NEC N=64	Control N=45	p
**gender**				0.509				0.706
**Female**	47 (45.6%)	13 (39.4%)	34 (48.6%)		57 (52.3%)	32 (50.0%)	25 (55.6%)	
**Male**	56 (54.4%)	20 (60.6%)	36 (51.4%)		52 (47.7%)	32 (50.0%)	20 (44.4%)	
**ethnicity:**				1.000				0.388
**African American**	70 (68.6%)	23 (69.7%)	47 (68.1%)		81 (75.7%)	50 (80.6%)	31 (68.9%)	
**Caucasian**	29 (28.4%)	9 (27.3%)	20 (29.0%)		17 (15.9%)	8 (12.9%)	9 (20.0%)	
**Other**	3 (2.94%)	1 (3.03%)	2 (2.90%)		9 (8.41%)	4 (6.45%)	5 (11.1%)	
**birthweight**	1150 [735;1617]	1140 [680;1540]	1185 [781;1620]	0.373	875 [650;1470]	735 [630;1128]	1020 [780;1680]	**0.010**
**gestational age**	29.1 [25.6;31.6]	29.2 [25.1;31.1]	29.1 [25.7;32.2]	0.567	27.0 [25.0;31.1]	26.5 [24.4;29.7]	28.6 [25.4;32.0]	**0.037**
**Mode of Delivery**				0.326				0.167
**C-Section**	73 (70.9%)	26 (78.8%)	47 (67.1%)		80 (74.1%)	51 (79.7%)	29 (65.9%)	
**Vaginal**	30 (29.1%)	7 (21.2%)	23 (32.9%)		28 (25.9%)	13 (20.3%)	15 (34.1%)	
**Chronic Hypertension**				0.297				0.442
**No**	79 (77.5%)	23 (69.7%)	56 (81.2%)		83 (77.6%)	51 (81.0%)	32 (72.7%)	
**Yes**	23 (22.5%)	10 (30.3%)	13 (18.8%)		24 (22.4%)	12 (19.0%)	12 (27.3%)	
**SGA**				0.137				**0.008**
**No**	84 (82.4%)	24 (72.7%)	60 (87.0%)		71 (67.0%)	34 (55.7%)	37 (82.2%)	
**Yes**	18 (17.6%)	9 (27.3%)	9 (13.0%)		35 (33.0%)	27 (44.3%)	8 (17.8%)	
**PIH**				**0.014**				**<0.001**
**No**	81 (79.4%)	21 (63.6%)	60 (87.0%)		80 (74.8%)	38 (60.3%)	42 (95.5%)	
**Yes**	21 (20.6%)	12 (36.4%)	9 (13.0%)		27 (25.2%)	25 (39.7%)	2 (4.55%)	
**chorioamnionitis**				1.000				0.488
**No**	87 (86.1%)	29 (87.9%)	58 (85.3%)		94 (87.9%)	57 (90.5%)	37 (84.1%)	
**Yes**	14 (13.9%)	4 (12.1%)	10 (14.7%)		13 (12.1%)	6 (9.52%)	7 (15.9%)	
**antenatal steroids:**				0.519				0.134
**No**	18 (19.8%)	8 (25.0%)	10 (16.9%)		24 (23.5%)	18 (29.5%)	6 (14.6%)	
**Yes**	73 (80.2%)	24 (75.0%)	49 (83.1%)		78 (76.5%)	43 (70.5%)	35 (85.4%)	
**Apgar <6 @5 minutes**				0.288				**0.022**
**No**	83 (83.8%)	30 (90.9%)	53 (80.3%)		78 (73.6%)	40 (64.5%)	38 (86.4%)	
**Yes**	16 (16.2%)	3 (9.09%)	13 (19.7%)		28 (26.4%)	22 (35.5%)	6 (13.6%)	
**disposition**				0.065				**0.004**
**Alive**	84 (82.4%)	31 (93.9%)	53 (76.8%)		86 (78.9%)	44 (68.8%)	42 (93.3%)	
**Dead**	18 (17.6%)	2 (6.06%)	16 (23.2%)		23 (21.1%)	20 (31.2%)	3 (6.67%)	
**Length of stay**	42.0 [17.0;89.0]	73.0 [41.0;126]	26.0 [6.75;69.8]	**<0.001**	88.0 [44.5;124]	103 [64.0;163]	73.0 [30.0;97.0]	**0.001**

**Table 2:** Clinical information for infants receiving antibiotics less than and more than 3 days

	Antibiotics =< 3days					Antibiotics >3days				
	[ALL] N=103	Medical NEC N=20	Surgical NEC N=13	Control N=70	P value	[ALL] N=109	Medical NEC N=34	Surgical NEC N=30	Control N=45	P value
**Male**	56 (54.4%)	12 (60.0%)	8 (61.5%)	36 (51.4%)	0.681	52 (47.7%)	18 (52.9%)	14 (46.7%)	20 (44.4%)	0.74
**ethnicity:**					0.952					0.784
**African American**	70 (68.6%)	14 (70.0%)	9 (69.2%)	47 (68.1%)		81 (75.7%)	27 (81.8%)	23 (79.3%)	31 (68.9%)	
**Caucasian**	29 (28.4%)	5 (25.0%)	4 (30.8%)	20 (29.0%)		17 (15.9%)	4 (12.1%)	4 (13.8%)	9 (20.0%)	
**Other**	3 (2.94%)	1 (5.00%)	0 (0.00%)	2 (2.90%)		9 (8.41%)	2 (6.06%)	2 (6.90%)	5 (11.1%)	
**Birth Weight in grams**	1150 [735;1617]	1415 [1008;1694]	710 [560;990]	1185 [781;1620]	**0.016**	875 [650;1470]	860 [652;1315]	700 [592;942]	1020 [780;1680]	**0.012**
**Gestational age in weeks**	29.1 [25.6;31.6]	30.3 [28.1;31.7]	25.0 [24.3;27.0]	29.1 [25.7;32.2]	**0.009**	27.0 [25.0;31.1]	27.2 [24.7;30.5]	26.4 [24.2;28.9]	28.6 [25.4;32.0]	0.062
**C-Section**	73 (70.9%)	16 (80.0%)	10 (76.9%)	47 (67.1%)	0.57	80 (74.1%)	30 (88.2%)	21 (70.0%)	29 (65.9%)	0.069
**Chronic Hypertension**	23 (22.5%)	5 (25.0%)	5 (38.5%)	13 (18.8%)	0.242	24 (22.4%)	6 (17.6%)	6 (20.7%)	12 (27.3%)	0.580
**SGA**	18 (17.6%)	6 (30.0%)	3 (23.1%)	9 (13.0%)	0.180	35 (33.0%)	12 (38.7%)	15 (50.0%)	8 (17.8%)	**0.011**
**PIH**	21 (20.6%)	8 (40.0%)	4 (30.8%)	9 (13.0%)	**0.021**	27 (25.2%)	13 (38.2%)	12 (41.4%)	2 (4.55%)	**<0.001**
**Chorioamnionitis**	14 (13.9%)	3 (15.0%)	1 (7.69%)	10 (14.7%)	0.915	13 (12.1%)	3 (8.82%)	3 (10.3%)	7 (15.9%)	0.702
**antenatal steroids:**	73 (80.2%)	16 (80.0%)	8 (66.7%)	49 (83.1%)	0.360	78 (76.5%)	24 (70.6%)	19 (70.4%)	35 (85.4%)	0.221
**Apgar <6 @ 5 mins**	16 (16.2%)	1 (5.00%)	2 (15.4%)	13 (19.7%)	0.376	28 (26.4%)	9 (27.3%)	13 (44.8%)	6 (13.6%)	**0.012**
**Mortality**	18 (17.6%)	0 (0.00%)	2 (15.4%)	16 (23.2%)	**0.035**	23 (21.1%)	8 (23.5%)	12 (40.0%)	3 (6.67%)	**0.002**
**Length of stay in days**	42.0 [17.0;89.0]	46.0 [38.2;77.0]	121 [94.0;175]	26.0 [6.75;69.8]	**<0.001**	88.0 [44.5;124]	90.0 [48.0;119]	148 [88.8;184]	73.0 [30.0;97.0]	**<0.001**

**Table 3 :** Clinical information in medical and surgical NEC cohort

	Medical NEC			Surgical NEC			
	[ALL] N=54	=<3 N=20	>3 N=34	[ALL] N=43	=<3 N=13	>3 N=30	P value
**Male**	30 (55.6%)	12 (60.0%)	18 (52.9%)	22 (51.2%)	8 (61.5%)	14 (46.7%)	0.57
**Ethnicity**							0.371
**African American**	41 (77.4%)	14 (70.0%)	27 (81.8%)	32 (76.2%)	9 (69.2%)	23 (79.3%)	
**Caucasian**	9 (17.0%)	5 (25.0%)	4 (12.1%)	8 (19.0%)	4 (30.8%)	4 (13.8%)	
**Other**	3 (5.66%)	1 (5.00%)	2 (6.06%)	2 (4.76%)	0 (0.00%)	2 (6.90%)	
**Birth weight**	995 [692;1635]	1415 [1008;1694]	860 [652;1315]	710 [570;970]	710 [560;990]	700 [592;942]	0.979
**Gestational age**	28.4 [25.4;31.2]	30.3 [28.1;31.7]	27.2 [24.7;30.5]	26.0 [24.2;28.4]	25.0 [24.3;27.0]	26.4 [24.2;28.9]	0.731
**C-Section**	46 (85.2%)	16 (80.0%)	30 (88.2%)	31 (72.1%)	10 (76.9%)	21 (70.0%)	0.72
**Chronic hypertension**	11 (20.4%)	5 (25.0%)	6 (17.6%)	11 (26.2%)	5 (38.5%)	6 (20.7%)	0.270
**Small of gestational age**	18 (35.3%)	6 (30.0%)	12 (38.7%)	18 (41.9%)	3 (23.1%)	15 (50.0%)	0.191
**Pregnancy induced Hypertension**	21 (38.9%)	8 (40.0%)	13 (38.2%)	16 (38.1%)	4 (30.8%)	12 (41.4%)	0.733
**chorioamnionitis**	6 (11.1%)	3 (15.0%)	3 (8.82%)	4 (9.52%)	1 (7.69%)	3 (10.3%)	1.000
**Antenatal steroids**	40 (74.1%)	16 (80.0%)	24 (70.6%)	27 (69.2%)	8 (66.7%)	19 (70.4%)	1.000
**Apgar <6 at 5 minutes**	10 (18.9%)	1 (5.00%)	9 (27.3%)	15 (35.7%)	2 (15.4%)	13 (44.8%)	0.089
**Disposition**							0.164
**Alive**	46 (85.2%)	20 (100%)	26 (76.5%)	29 (67.4%)	11 (84.6%)	18 (60.0%)	
**Dead**	8 (14.8%)	0 (0.00%)	8 (23.5%)	14 (32.6%)	2 (15.4%)	12 (40.0%)	
**length of stay**	75.0 [40.0;103]	46.0 [38.2;77.0]	90.0 [48.0;119]	128 [89.5;183]	121 [94.0;175]	148 [88.8;184]	0.958

**Table 4:** Association between antibiotic duration (>3 days vs =< 3 days) and severity of NEC using binary logistic regression.

Predictor	*aOR*	*95% CI*	*P value*
NEC Severity			
Control	Ref.		
Surgical NEC	3.33	1.57–7.40	0.002
Medical NEC	2.61	1.35–5.16	0.005
Gestational age	0.98	0.90–1.06	0.536
Death	1.07	0.52–2.23	0.856

Note: Sample size 211.

Reference level of antibiotic duration was set as =<3 days.

**Table 4A:** Association between antibiotic duration (>3 days vs =< 3 days) and severity of NEC among the NEC patients using binary logistic regression.

Predictor	*aOR*	*95% CI*	*P value*
NEC Severity			
Medical NEC	Ref.		
Surgical NEC	0.88	0.33 – 2.28	0.787
Gestational age	0.92	0.81 – 1.05	0.211
Death	7.88	1.99 – 53.74	0.010

Note: Sample size 97.

Reference level of antibiotic duration was set as =<3 days.

**Table 5:** Distribution of Placental lesions in infants receiving antibiotics >and =<3 DAYS

	Antibiotic duration =< 3 days			Antibiotic duration > 3 days		
Combination	Medical NEC (n=20)	Surgical NEC (n=13)	Control (n=70)	Medical NEC (n=34)	Surgical NEC (n=30)	Control (n=45)
AHC + VE	0 (0%)	1 (7.7%)	3 (4.3%)	0 (0%)	1 (3.3%)	3 (6.7%)
AHC + CFR	2 (10%)	8 (61.5%)	8 (11.4%)	2 (5.9%)	8 (26.7%)	8 (17.8%)
AHC + MVM	4 (20%)	6 (46.2%)	8 (11.4%)	4 (11.8%)	6 (20%)	8 (17.8%)
MVM + CFR	2 (10%)	6 (46.2%)	3 (4.3%)	2 (5.9%)	6 (20%)	3 (6.7%)
MVM + VE	2 (10%)	1 (7.7%)	6 (8.6%)	2 (5.9%)	1 (3.3%)	6 (13.3%)
MVM + SGA	5 (25%)	1 (7.7%)	9 (12.9%)	5 (14.7%)	1 (3.3%)	9 (20%)
VE + SGA	0 (0%)	0 (0%)	4 (5.7%)	0 (0%)	0 (0%)	4 (8.9%)

AHC = acute histologic chorioamnionitis, VE= Villous edema, CFR = Chorioamnionitis with fetal response, MVM =maternal vascular malformation, GA= small of gestational age

**Table 6:** Placental pathologies and antibiotic exposure

	Overall	Medical NEC	Surgical NEC	Control	p-value
**Acute Histologic Chorio**	N=64	N=19	N=21	N=24	
Antibiotic duration:					**0.021**
≤ 3 days	27 (42%)	4 (21%)	8 (38%)	15 (63%)	
> 3 days	37 (58%)	15 (79%)	13 (62%)	9 (38%)	
Antibiotic duration:					0.06
≤ 5 days	30 (47%)	5 (26%)	10 (48%)	15 (63%)	
> 5 days	34 (53%)	14 (74%)	11 (52%)	9 (38%)	
**Chorio w Fetal Response**	N=40	N=9	N = 17	N=14	
Antibiotic duration:					0.3
≤ 3 days	18 (45%)	2 (22%)	8 (47%)	8 (57%)	
> 3 days	22 (55%)	7 (78%)	9 (53%)	6 (43%)	
Antibiotic duration:					0.6
≤ 5 days	20 (50%)	3 (33%)	9 (53%)	8 (57%)	
> 5 days	20 (50%)	6 (67%)	8 (47%)	6 (43%)	
**Maternal Vascular Malperfusion**	N=40	N = 12	N=7	N=21	
Antibiotic duration:					**0.007**
≤ 3 days	66 (49%)	17 (40%)	10 (33%)	39 (64%)	
> 3 days	68 (51%)	26 (60%)	20 (67%)	22 (36%)	
Antibiotic duration:					**0.001**
≤ 5 days	93 (69%)	25 (58%)	16 (53%)	52 (85%)	
> 5 days	41 (31%)	18 (42%)	14 (47%)	9 (15%)	
**SGA Placenta**	N=40	N = 12	N=7	N=21	
Antibiotic duration:					0.4
≤ 3 days	16 (40%)	5 (42%)	1 (14%)	10 (48%)	
> 3 days	24 (60%)	7 (58%)	6 (86%)	11 (52%)	
Antibiotic duration:					0.06
≤ 5 days	28 (70%)	6 (50%)	4 (57%)	18 (86%)	
> 5 days	12 (30%)	6 (50%)	3 (43%)	3 (14%)	
